# Detecting Analogies Unconsciously

**DOI:** 10.3389/fnbeh.2014.00009

**Published:** 2014-01-22

**Authors:** Thomas P. Reber, Roger Luechinger, Peter Boesiger, Katharina Henke

**Affiliations:** ^1^Department of Psychology, University of Bern, Bern, Switzerland; ^2^Center for Cognition, Learning and Memory, University of Bern, Bern, Switzerland; ^3^Institute for Biomedical Engineering, ETH Zurich, Zurich, Switzerland

**Keywords:** episodic memory, subliminal, analogical mapping, consciousness, flexibility, hippocampus, medial temporal lobe

## Abstract

Analogies may arise from the conscious detection of similarities between a present and a past situation. In this functional magnetic resonance imaging study, we tested whether young volunteers would detect analogies unconsciously between a current supraliminal (visible) and a past subliminal (invisible) situation. The subliminal encoding of the past situation precludes awareness of analogy detection in the current situation. First, participants encoded subliminal pairs of unrelated words in either one or nine encoding trials. Later, they judged the semantic fit of supraliminally presented new words that either retained a previously encoded semantic relation (“analog”) or not (“broken analog”). Words in analogs versus broken analogs were judged closer semantically, which indicates unconscious analogy detection. Hippocampal activity associated with subliminal encoding correlated with the behavioral measure of unconscious analogy detection. Analogs versus broken analogs were processed with reduced prefrontal but enhanced medial temporal activity. We conclude that analogous episodes can be detected even unconsciously drawing on the episodic memory network.

## Introduction

We pull up analogies to make inventions, solve problems, and plan and adapt our behavior in new situations. The detection of analogies assumes a source situation that is remembered and a present situation that is interpreted in light of the source situation (Gentner, 1983). For example, bodily and facial cues of a young couple flirting might suddenly remind us of an episode at a scientific session where two scientists eager for collaboration made their first contact. Analogies arise from the detection of correspondences or *mappings* between elements in a current situation and elements in a memory representation of a past situation. The detection of situational analogies depends on a flexible expression of source memories because the format and elements of the source situation often differ from the format and elements of a current situation. Flexibility of memory expression is considered a hallmark of episodic memory (Cohen and Eichenbaum, 1993; Frank et al., 2003; Henke, 2010).

Analogies often spring to mind suddenly and unexpectedly. The detection of analogies likely transcends an unconscious stage before entering consciousness. The source knowledge is usually stored in either episodic (Gentner et al., 1993; Schunn and Dunbar, 1996; Wharton et al., 1996; Day and Goldstone, 2011) or semantic memory (Spellman et al., 2001; Bunge et al., 2005; Green et al., 2006a,b, 2010) with both forms of memory hypothesized to be associated with consciousness of encoding/retrieval (Squire and Zola, 1996; Moscovitch, 2008). Episodic memory depends on hippocampal processing (Cohen and Eichenbaum, 1993; Reber and Squire, 1994; Squire and Zola, 1996; Squire et al., 2007; Moscovitch, 2008) and hippocampal activity closely tracks conscious experience (Kreiman et al., 2002; Quiroga et al., 2008). A prominent role in analogical mapping has been assigned to the prefrontal cortex (Morrison et al., 2004; Bunge et al., 2005; Green et al., 2006b, 2010; Speed, 2010; Knowlton et al., 2012), which has also been linked to consciousness of information processing (Dehaene and Naccache, 2001; Dehaene and Changeux, 2011). Hence, the detection of analogies in a current situation may require the conscious retrieval of a source situation.

We hypothesize that the detection of analogies between a source and a current situation does not strictly require conscious awareness of encoding/retrieving. On the contrary, we assume that analogical mapping proceeds automatically and unconsciously as a consequence of a facilitated processing of relations between elements in a current situation based on the past experience of similar relations (Schunn and Dunbar, 1996; Leech et al., 2008). Then, unconscious analogical mapping may or may not emerge to consciousness. Evidence for unconscious analogical mapping comes from purely behavioral studies that assessed awareness of analogical mapping with *post hoc* self-reports (Schunn and Dunbar, 1996; Silberman et al., 2005; Green et al., 2006a; Day and Gentner, 2007; Day and Goldstone, 2011). A more stringent way to test for unconscious analogical mapping is by presenting the source situation subliminally (invisibly) for unconscious encoding. Using subliminal presentations and functional magnetic resonance imaging (fMRI), we gained evidence that unconscious analogy detection is feasible by the way of episodic memory network including hippocampus.

## Materials and Methods

### Overview

**Figure 1:**
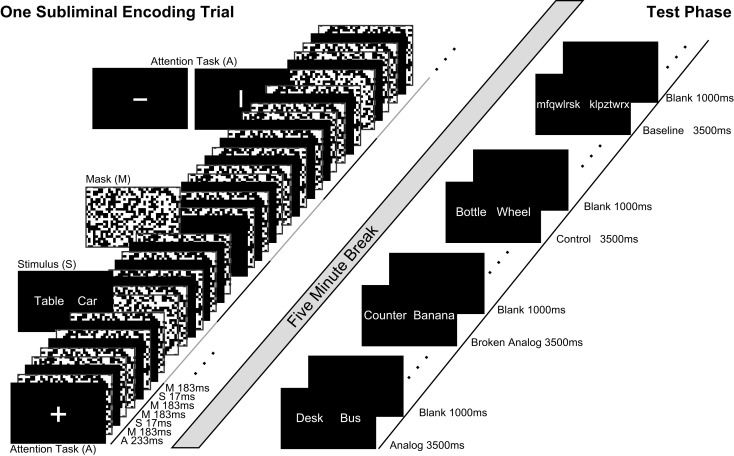
**Subliminal and supraliminal trials**. Left side, subliminal word pairs were presented for 17 ms between pattern masks. Participants were not informed of subliminal stimuli; they performed an attention task during the subliminal stimulation sequence. There was a break of 5 min between encoding and test. Right side, at test, pairs of new words were presented supraliminal in three conditions for participants to decide whether words in a pair fit together semantically. Words in analogs were new but retained semantic relations from subliminal encoding word pairs (encoding: table–car; test: desk–bus). Semantic relations were broken in Broken Analogs. Control pairs consisted of words that were neither semantically close to encoding words nor retained semantic relations. Successive stimuli are depicted from bottom left to top right.

**Figure 2:**
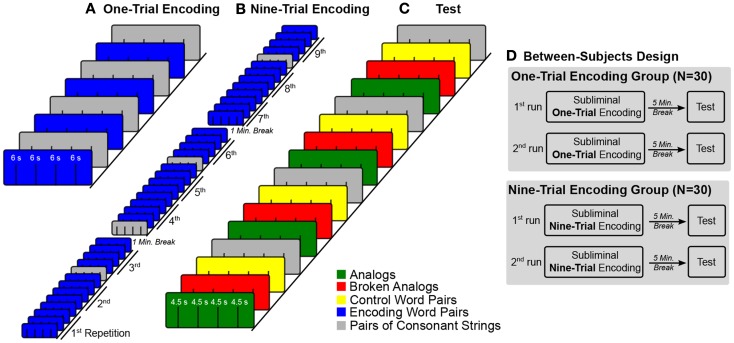
**Experimental design**. **(A–C)** The colored boxes illustrate trial blocks that belong to a certain experimental condition. Lines within colored boxes stand for individual trials. The sequence of blocks over time is given from bottom left to top right. **(A)** The fMRI time-series for subliminal one-trial encoding entailed four blocks of four subliminal word pairs each and four blocks of four pairs of consonant strings each. Blocks of word pairs (blue) alternated with blocks of pairs of consonant strings (gray). **(B)** In the fMRI time-series for subliminal nine-trial encoding, the four blocks with word pairs were repeated nine times for a better encoding. Blocks with pairs of consonant strings were not repeated. These blocks were interspersed at pseudo-random positions in the time-series. **(C)** The test fMRI time-series embraced four conditions of four blocks each. Each block contained four trials. Condition blocks were presented in a fixed alternating sequence. **(D)** Half of participants were assigned to the one-trial encoding group and the other half to the nine-trial encoding group. Both groups took two experimental runs. Each run consisted of an encoding part, a 5-min encoding-test interval, and a test part.

For subliminal encoding, we presented pairs of unrelated words (e.g., table–car) to establish novel unconscious source knowledge (Figure 1). Participants performed an attention task during subliminal encoding to ensure that their attentional focus remained on the stimulus display. After 5 min of quiet rest, we presented word pairs for conscious inspection that consisted of new (not subliminally presented) words that were conceptually related to the subliminal encoding words (Figure 1). The conceptual relations that were established during unconscious encoding were either kept intact at test (analogs) or not (broken analogs) (Silberman et al., 2005; Reber and Henke, 2011). Thus, an analogous word pair (e.g., desk–bus) maintained the conceptual relation (e.g., a piece of furniture–a means of transport) that was established during an encoding trial (e.g., table–car). Broken analogs combined words (e.g., counter–banana) that were conceptually related to words from two different encoding trials (e.g., table–car; keyboard–apple). The participants’ task was to judge whether the two words in a pair fit together semantically or not (forced-choice test). More fit responses to analogs than broken analogs suggest that novel source knowledge was established and that the mapping of source knowledge onto the target was successful. Crucially, the participants were unaware of any memory reactivation because encoding stimuli were subliminal. Subliminal encoding precluded participants’ awareness of analogical relations between encoding and test words. We varied the number of encoding trials between participants to measure potential effects of subliminal overlearning such as semanticization (disengagement of hippocampus over encoding trials) and unitized versus compositional representation of encoding word pairs. To this aim, half of participants (*N* = 30) encoded each subliminal word pair in one subliminal encoding trial, while the other half of participants encoded each subliminal word pair in nine subliminal encoding trials (Figure 2D).

### Participants

Participants were 60 right-handed men with a mean age of 24.6 years [standard deviation (SD) = 4.6 years]. They reported no current or past neurological or psychiatric illness, were native German speakers, and had normal or corrected-to-normal vision. The study was approved by the local ethics committee.

The analysis of the behavioral data included the datasets of 57 participants. Three participants were excluded because their performance on the attention task (given during subliminal stimulation) was below two SDs of the group mean. SDs were derived separately for the participants in the one-trial encoding condition and participants in the nine-trial encoding condition because mean hit rates on the attention task differed between encoding conditions (see Results).

Performance on the attention task correlated inversely with the quality of encoding subliminal word pairs (see Results). Hence, performance on the attention task could be taken as proxy for subliminal encoding quality. In order to isolate the good from the poor subliminal encoders, we performed a median split on the *z*-values of the attention task. In the following, we refer to low versus high performers on the attention task as good versus poor subliminal encoders, respectively. Because half of participants encoded word pairs in one and half in the nine subliminal encoding trials, the median split resulted in four subgroups of participants: of 29 poor subliminal encoders, 15 were in the one-trial encoding condition and 14 in the nine-trial encoding condition; of 28 good subliminal encoders, 13 were in the one-trial encoding condition and 15 in the nine-trial encoding condition.

The analysis of functional imaging data included 51 participants as the data of nine participants were excluded because of the excessive scan-to-scan movements (*N* = 1), slice-artifacts (*N* = 5), and – as mentioned above – very low performance on the attention task (*N* = 3). Of the 51 evaluated fMRI datasets, 27 corresponded to poor subliminal encoders, of whom 14 belonged to the one-trial encoding condition and 13 to the nine-trial encoding condition. Twenty-four datasets corresponded to good subliminal encoders, of whom 12 were in the one-trial encoding condition and 12 in the nine-trial encoding condition.

### Apparatus

The experiment took place in a magnetic resonance imaging (MRI) chamber that was darkened by turning off all lights and by a black curtain that prevented light from entering the MRI chamber. A BenQ SP831 DLP projector was placed in between the curtain and the shielding glass to project the stimulus sequence onto a screen positioned in front of the MR scanner. Stimuli spanned a visual field of 11°(height) × 13 (width). They were back-projected onto the screen. Participants viewed the stimuli through two mirrors mounted on the head-coil. The stimulus sequence was generated by a laptop running the software Presentation^1^. The visual display had a resolution of 1024 × 768 pixel and was presented with a refresh rate of 60 Hz.

### Subliminal presentation protocol

One subliminal encoding trial entailed 12 presentations of a word pair (W) within a 6-s time-window (Degonda et al., 2005) (Figure 1). Each of the 12 presentations lasted 17 ms and was preceded and followed by random-dot pattern masks (M; masking stimuli) that were presented for 183 ms each. Preceding two such masked presentations of a word pair (M W M M W M), either a fixation cross, a horizontal, or a vertical bar (A) was presented for 233 ms. This presentation sequence (A M W M M W M) lasted 1 s and was repeated six times with the same word pair as subliminal stimulus. The fixation cross was presented randomly five in six times; a horizontal or vertical bar was presented with equal probability once in six times. The participants’ task was to focus gaze on the fixation cross/bar and to indicate the orientation of a bar by button press immediately upon the bar’s occurrence.

### Practice run

The practice run allowed for the conscious inspection of both encoding and test word pairs. We wanted participants to note the correspondence between encoding and test word pairs, which should allow them to install a task-set for the ensuing subliminal trials. Task-sets may guide the processing of subliminal stimuli (Kiefer and Martens, 2010; Reber and Henke, 2011). The encoding part of the practice run took 2.4 min. It was followed by 5 min of rest. The practice run ended with the test part, which took 4.8 min. Encoding and test word pairs were presented once for 3.5 s with inter-stimulus intervals of 1 s. Participants engaged in the same forced-choice task during encoding and test. The forced-choice task required them to decide whether the two words in an encoding or test pair fit together semantically or not. Since words in all pairs were semantically distant, we asked participants to relax their response criterion in order to give an approximately equal amount of fit and don’t fit responses. When pairs of consonant strings (baseline) were presented, participants were asked to judge the visual fit between two consonant strings as if they were two art sculptures. This instruction was chosen to foster a holistic processing of consonant strings, which makes the processing comparable to the equally holistic processing of words in pairs.

After the completion of this supraliminal practice run, participants were interviewed on whether they had noticed the correspondence between encoding word pairs and analogs. If they could name at least one encoding word pair and its analog (e.g., table–car; desk–bus), they were classified as having gained insight into the task structure. In a previous study (Reber and Henke, 2011), participants with versus without insight performed better in the following subliminal encoding and retrieval task. This finding was not replicated in the present study. Twenty-eight participants gained insight into the task structure during the practice run. The difference in the baseline-corrected percentage of fit responses given to analogs versus broken analogs in the main experiment did not differ between participants, who gained insight (*M* = 2.4%, SD = 1.2%, *N* = 28), and participants, who failed to gain insight (*M* = 0.5%, SD = 1.1%, *N* = 29), *t*(55) = 0.625, *p* = 0.534.

### Main experiment

The main experiment consisted of two experimental runs. Each experimental run started with the encoding part, included a 5-min break and ended with the test part. Unlike the practice run, encoding word pairs were presented subliminally for unconscious encoding (Figure 1). The task at test was again a forced-choice judgment of the semantic fit between the two supraliminal words in a test pair, as in the practice run.

For half of the participants, encoding word pairs were presented in one subliminal encoding trial (one-trial encoding) during both experimental runs. Encoding and test word pairs were presented in blocks of four with four blocks per condition (Figure 2A). Blocks of encoding word pairs alternated with blocks of pairs of consonant strings (baseline). Half of the encoding time-series started with a block of word pairs and half with a block of pairs of consonant strings. There was a 5-min break between the encoding and test fMRI time-series. In the test fMRI time-series, the order of condition blocks (conditions: analogs, broken analogs, control word pairs, and pairs of consonant strings) was varied between participants according to a Latin-square (Figure 2C). The fMRI time-series on subliminal one-trial encoding took 3.2 min and the test fMRI time-series took 4.8 min.

For the other half of participants, each encoding word pair was presented in nine temporally dispersed encoding trials (nine-trial encoding) in both experimental runs (Figure 2B). To alleviate tiring, nine-trial encoding was split into three encoding fMRI time-series per experimental run, separated by 1 min breaks, during which time no fMRI data were acquired. The ninefold repetition concerned only word pairs presented in the experimental condition but not the pairs of consonant strings presented in the baseline condition – these were shown only once. The four condition blocks that contained pairs of consonant strings were pseudo-randomly intermixed with the condition blocks that contained word pairs. Before any block of word pairs was repeated, we presented the complete set of encoding word pairs. At test, the order of condition blocks (conditions: analogs, broken analogs, control word pairs, and pairs of consonant strings) was varied between participants according to a Latin-square (Figure 2C). The three fMRI time-series on subliminal encoding over nine-trials lasted 16 min in total (5.3 min per times-series; no scanning during the 1-min breaks between time-series). The test fMRI time-series took 4.8 min.

### Awareness test

After the main experiment, participants were asked whether they had noticed words or perceptual fragments thereof during subliminal encoding. Then, participants were informed of the masked presentation of subliminal stimuli. Finally, we conducted an awareness test to measure participants’ ability to discriminate masked words. This awareness test consisted of 30 encoding-test trials. On each trial, a word was presented in one subliminal encoding trial with the masking technique of the main experiment. Immediately following the subliminal presentation of a word, we presented two supraliminal words side-by-side for participants to do a forced-choice task. Participants chose which word was semantically related to the preceding subliminal word. The target word was semantically related to the subliminal word and the distracter word was unrelated. The side of the target/distracter was randomized.

### Stimuli

We assembled 192 triplets of words that consisted of subordinates to the same concept (e.g., table–desk–counter; car–bus–truck; apple–pear–banana). These triplets were assigned to six lists each containing 32 triplets. Two lists were assigned to the practice run, two lists to the first experimental run, and two lists to the second experimental run. For each run, one list was used to create encoding word pairs, analogs, and broken analogs. The other list was used to create the control word pairs presented at test only. Encoding word pairs were formed by combining the first words of two different triplets (e.g., table–car). Analogs were formed by combining the second words of these triplets (e.g., desk–bus) and broken analogs by combining the third words of two triplets (e.g., counter–banana). Control word pairs were constructed by combining the first, the second, or the third words in two triplets. Furthermore, pairs of randomly generated consonant strings (e.g., cvmgwpls–pklwqvcn; eight different consonants per string) were presented in the encoding and test time-series as a baseline condition. A stimulus list entailed 16 pairs of words or consonant strings (conditions of the encoding fMRI time-series: encoding word pairs, pairs of consonant strings; conditions of the retrieval fMRI time-series: analogs, broken analogs, control word pairs, and pairs of consonant strings).

To counterbalance word pairs between the analogs and broken analogs condition, encoding word pairs were re-arranged (e.g., original list: table–car; counterbalanced list: table–apple). The resulting analogs (counter–pear) and broken analogs (desk–bus) were thus identical to broken analogs and analogs, respectively, in the initial arrangement. The assignment of the six lists of triplets to conditions and experimental runs was balanced between participants. A further list of stimuli was used for the test of awareness, which was conducted after the main experiment. For the test of awareness, we compiled 60 pairs of conceptually related words. Two pairs (four words) were assigned to one-trial in the awareness test. The word used for subliminal presentation was randomly chosen from the four words. The semantically related word was the target and the distracter word was randomly chosen from the two remaining words.

### Magnetic resonance imaging

The experiment was conducted on a 1.5-T Philips whole-body MRI scanner. We used an eight-channel head coil. fMRI data were obtained with a sensitivity-encoded single-shot echo planar imaging sequence with an acceleration factor *r* = 2.0 (Schmidt et al., 2005). Thirty-four slices along the AC–PC line were acquired without inter-slice gaps. The time of repetition (TR) was 3 s, echo-time (TE) 50 ms, flip-angle θ = 90°. The field of view was 22 cm × 22 cm. The measured voxel-size was 2.75 mm × 2.75 mm × 4 mm, which was reconstructed to a voxel-size of 1.72 mm × 1.72 mm × 4 mm. A standard 3D T1 image was acquired as anatomical reference (TE = 3.8 ms, TR = 8.2 ms, flip-angle θ = 8°, 160 slices, original voxel-size = 1 mm × 1 mm × 1 mm, no interpolation, field of view = 24 cm × 24 cm, no inter-slice gaps).

### Analysis of functional magnetic resonance imaging data

The fMRI data were analyzed using the statistical parametric mapping toolbox^2^ (SPM 8). Preprocessing included spatial realignment of the EPI images, normalization of EPI images to a standard anatomical space (mean image of 152 subjects from the Montreal Neurological Institute), and spatial smoothing with an 8-mm Gaussian kernel.

fMRI scanning was halted during the breaks between encoding and test.

First-level models were estimated separately for encoding and test. These models included regressors that were created by convolving a canonical hemodynamic response function with box-car functions of the on- and off-sets of the experimental conditions, and six movement-regressors that were estimated during spatial realignment. First-level contrast images of interest (e.g., pairs of words > pairs of consonant strings) were subjected to random effects analyses. Random effects analyses were thresholded at *p* = 0.001 (uncorrected), and the minimum cluster extent was set to 20 consecutive voxels. We adopted a looser statistical threshold (*p* = 0.005, no cluster extent) for the medial and anterior temporal lobe because these regions are of particular interest in the current study.

We needed to compute separate models for one-trial and nine-trial encoding because the encoding time-series differed (Figure 2). Hence, we computed separate second-level analyses for one-trial and nine-trial encoding. Based on the behavioral data, we split participants into good and poor subliminal encoders. The second-level analyses therefore included the between-subjects factor Encoding Quality (good versus poor subliminal encoders). The contrast of one-trial encoding was pairs of words > pairs of consonant strings including the data of the good subliminal encoders (*N* = 12). The contrast of nine-trial encoding contained beta-weights of the linear increase of the BOLD signal over the nine repetitions of subliminal word pairs including the data of the good subliminal encoders (*N* = 12). To investigate neural activity obtained at test, we computed an ANOVA with the within-subjects factor Test Condition (analogs, broken analogs) and the between-subjects factors Learning Intensity (one-trial encoding, nine-trial encoding) and Encoding Quality (good subliminal encoders, poor subliminal encoders). The dependent measure consisted of the first-level contrast images analogs > control word pairs and broken analogs > control word pairs. The reported analysis is a *t*-test of analogs versus broken analogs of this ANOVA for the good subliminal encoders. This contrast collapsed data over Learning Intensity because Learning Intensity did not influence behavioral results (*N* = 24).

To investigate brain–behavior correlations, we performed one-sample *t*-tests of contrasts of interest (e.g., analogs > broken analogs) including the behavioral measure obtained at test as a covariate of interest.

### Analysis of behavioral data

**Figure 3:**
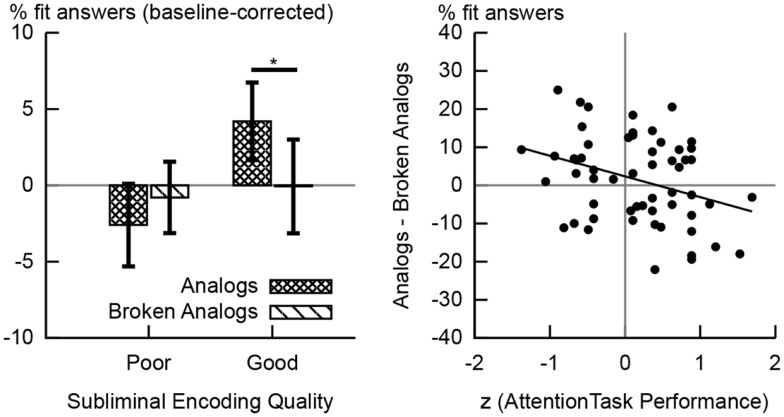
**Behavioral results**. The left-hand chart shows the percentages of fit responses given to analogs and broken analogs by the group of good and the group of poor subliminal encoders. These percentage values are baseline-corrected; i.e., the percentage of fit responses given to control word pairs was subtracted. Error bars indicate standard errors of means. The difference in the percentage of fit responses to analogs versus broken analogs reached significance at the 5% level in good subliminal encoders. The right-hand chart shows the scatter plot of the correlation between performance on the attention task (during subliminal encoding) and unconscious analogy detection at test (difference in the percentage of fit responses to analogs versus broken analogs). A poorer performance on the attention task predicted a better performance at detecting analogies (*R* = −0.332,*p* = 0.012).

Reaction times (RTs) of semantic fit responses given at test were *z*-transformed per participant. Trials with *z*-values below or above *M* ± 2 SD were excluded from analysis. Data were aggregated per condition (mean of RTs; sum of fit/don’t fit responses). We arrived at the percentage of fit responses by dividing the number of fit responses given in a certain condition (e.g., analogs, broken analogs, control word pairs) by the sum of fit and don’t fit responses given in that condition. To control for a subjects’ generic propensity to give fit responses, the percentage of fit responses to control word pairs was subtracted from both the percentage of fit responses given to analogs and the percentage of fit responses given to broken analogs. We refer to the resulting numbers as baseline-corrected percentages of fit responses (Figure 3).

## Results

### Good performance on the attention task

Because nine-trial encoding took longer and was more tiring than one-trial encoding, the rate of correct responses on the attention task was lower in the nine-trial (*M* = 80%, SD = 2%, *N* = 29) than the one-trial encoding condition (*M* = 96%, SD = 3%, *N* = 28) [*t*(55) = 21.066, *p* < 10^−27^]. Still, the rates of correct responses were high enough in both conditions to assume that participants kept attention up throughout the experiment.

### Detection of analogies to unconscious memories

We had hypothesized that the unconscious detection of analogies would yield more fit responses to analogs than broken analogs (Reber and Henke, 2011). Surprisingly, no such effect was apparent for the whole sample (*N* = 57). However, performance on the attention task during subliminal encoding predicted whether participants could detect analogies and yielded a larger number of fit responses to analogs versus broken analogs. A poorer performance on the attention task predicted more fit responses to analogs than broken analogs in the whole sample (*R* = −0.332, *p* = 0.012, *N* = 57, Figure 3). It thus appears that focusing too much on the attention task took away from the simultaneous processing of subliminal word pairs. We divided our sample by median split into low versus high performers on the attention task or good versus poor subliminal encoders, respectively. Good subliminal encoders gave more fit responses to analogs (baseline-corrected; *M* = 4.2%, SD = 13.5%; Figure 3) than to broken analogs (*M* = −0.6%, SD = 16.3%), *t*(27) = 2.432, *p* = 0.022 (Figure 3). No such difference was found for poor subliminal encoders [*M*_analogs_ = −2.6%, SD_analogs_ = 14.5%, *M*_broken analogs_ = −0.8%, SD_broken analogs_ = 12.6%, *t*(28) = −0.858, *p* = 0.398; Figure 3].

The number of encoding trials did not influence performance at test. The number of fit responses given to analogs versus broken analogs was statistically equal between the one-trial and the nine-trial encoding condition: (a) across the whole group of participants [*t*(55) = −0.311, *p* = 0.757], (b) within the group good subliminal encoders [*t*(26) = −0.907, *p* = 0.373], and (c) within the group of poor subliminal encoders [*t*(27) = 0.549, *p* = 0.588]. This and previous findings (Reber and Henke, 2011) bolster the view that young and healthy participants encode subliminal word pairs equally well in one and nine trials.

### Participants were unaware of subliminal stimuli

Interviews conducted after the main experiment revealed that no participant had noticed words or perceptual fragments thereof during subliminal encoding. The test of awareness indicated that participants were unable to discern the subliminal stimuli when directly instructed to do so. The mean frequency of correct responses (*M* = 14.98, SD = 2.35) did not differ from the chance performance (15 correct responses); *t*(56) = −0.056, *p* = 0.955. Neither was there a significant difference in the number of correct responses between the participants in the nine-trial versus the one-trial encoding condition [*M*_9 trial_ = 15.45, SD_9 trial_ = 2.84, *M*_1 trial_ = 14.50, SD_1 trial_ = 1.62; *t*(55) = −1.542, *p* = 0.129] nor between good versus poor subliminal encoders [*M*_good encoders_ = 15.05, SD_good encoders_ = 2.40, *M*_poor encoders_ = 14.93, SD_poor encoders_ = 2.34; *t*(55) = 0.167, *p* = 0.868].

### Unconscious encoding of word pairs recruits regions of the episodic memory network

Based on previous findings (Henke et al., 2003a,b; Degonda et al., 2005; Reber et al., 2012), we hypothesized that structures of the episodic memory network including the hippocampus would support one-trial encoding of subliminal word pairs. To address this hypothesis, we tested for increases of brain activity during blocks of subliminal word pairs versus blocks of subliminal pairs of consonant strings presented in runs with one encoding trial. We are reporting fMRI results for the good subliminal encoders because only the group of good subliminal encoders showed a successful unconscious detection of analogies as indicated by a larger number of fit responses given to analogs than to broken analogs (Figure 3). Significant activity increases were located in two regions within the left hippocampus (Table 1). A further cluster of increased activity emerged in the precuneus (BA 7), a region that has been implicated in the mnemonic processing of items with rich contextual details (Gilboa et al., 2004; Cavanna and Trimble, 2006). Further activity increases were located in the posterior cingulate gyrus (BA 31), a constituent of the episodic memory network (Cabeza and Nyberg, 2000), and in the inferior frontal gyrus (BA 46), which has been implicated in the encoding of the relationships between two elements in an associative stimulus (Blumenfeld and Ranganath, 2006; Murray and Ranganath, 2007). Further activity increases targeted BA 6 including the medial frontal gyrus and the precentral gyrus; both regions are thought to support a fluent semantic retrieval (Chee et al., 1999; Binder et al., 2009; Graves et al., 2010; Segaert et al., 2012).

**Table 1:** **Onefold encoding of subliminal word pairs**.

Brain region	BA	Left/right	MNI-coordinates			*k*	*t*	*z*
			*x*	*y*	*z*			
**WORD PAIRS > CONSONANT STRINGS**								
Inferior frontal gyrus	46	R	40	34	8	21	4.17	3.56
Medial frontal gyrus	6	L/R	0	−10	70	121	4.77	3.94
Precentral gyrus	6	L	−60	−12	40	21	4.62	3.85
Hippocampus		L	−38	−22	−12	55	4.22	3.59
Hippocampus		L	−32	−40	0	59	4.42	3.72
Posterior cingulate gyrus	31	L	−10	−44	46	33	4.93	4.03
Precuneus	7	R	8	−60	44	49	5.03	4.09
**CONSONANT STRINGS > WORD PAIRS**								
Lingual gyrus, fusiform gyrus	18	L	−22	−78	−6	38	5.00	4.07

**N* = 12 (good subliminal encoders); statistical maps thresholded at *p* = 0.005, *k* = 0 in medial and anterior temporal lobe regions, all other regions *p* = 0.001, *k* = 20*.

The analyses of nine-trial subliminal encoding corroborate that structures of the episodic memory network subserved the encoding of subliminal word pairs. We assessed effects of a regressor that increased linearly with each repetition of a subliminal word pair (Table 2). Good subliminal encoders displayed signal increases over repetitions in the left anterior hippocampus and the right hippocampus extending into right rhinal cortex. Although stimulus repetitions have been reported to be associated with decreasing activity (repetition suppression) in the medial temporal lobe (Turk-Browne et al., 2006; Vannini et al., 2012; Manelis et al., 2013), others report repetition enhancement (Kirwan et al., 2009; Greene and Soto, 2012). A likely reason for the current repetition enhancement is the brief presentation time (17 ms), which is associated with a weak signal. But this weak signal may gain in strength over repetitions, an effect found in a previous study (Müller et al., 2013). A progressively deeper semantic analysis of word pairs was suggested by a linear increase of activity in lateral temporal cortex and prefrontal cortex. Temporal repetition enhancement was located in the bilateral temporal poles and left inferior temporal gyrus suggesting that words were analyzed to a high level of abstraction (Patterson et al., 2007). Prefrontal repetition enhancements were located in the medial and superior frontal gyrus (both BA 6), which have been implicated in fluent semantic retrieval (Binder et al., 2009). There were also two regions of repetition suppression, one in left middle occipital gyrus and the other in left supramarginal gyrus likely associated with a facilitated visual analysis of words (Stoeckel et al., 2009).

**Table 2:** **Ninefold encoding of subliminal word pairs**.

Brain region	BA	Left/right	MNI-coordinates			*k*	*t*	*z*
			*x*	*y*	*z*			
**LINEAR INCREASE OF BOLD SIGNAL WITH STIMULUS REPETITIONS**								
Temporal pole	38	R	38	22	−38	80	5.91	4.53
Temporal pole	38	L	−36	18	−40	37	4.73	3.89
Superior frontal gyrus	6	L	−12	16	68	24	4.41	3.7
Inferior temporal gyrus	20	L	−52	−4	−36	57	6.19	4.66
Putamen		R	26	−12	4	29	4.58	3.8
Hippocampus		L	−28	−14	−24	69	4.36	3.66
Hippocampus, rhinal cortex	35	R	26	−22	−18	27	4.42	3.7
Medial frontal gyrus	6	L/R	8	−28	62	72	5.53	4.33
**LINEAR DECREASE OF BOLD SIGNAL WITH STIMULUS REPETITIONS**								
Middle occipital gyrus	19	L	−32	−92	26	40	5.46	4.29
Supramarginal gyrus	40	L	−62	−48	32	37	4.79	3.92

**N* = 12 (good subliminal encoders); statistical maps thresholded at *p* = 0.005, *k* = 0 in medial and anterior temporal lobe regions, all other regions *p* = 0.001, *k* = 20*.

### Facilitated processing of semantic relations in analogs

To assess neural correlates of unconscious analogy detection, we contrasted neural activity of good subliminal encoders between the processing of analogs and broken analogs (collapsed over Learning Intensity) (Table 3). Analogs versus broken analogs evoked activity enhancements in the left perirhinal cortex. Large areas in the prefrontal cortex exhibited reduced activity during the processing of analogs versus broken analogs (Table 3). We assume that memories of subliminal word pairs were reactivated through the left perirhinal cortex. The perirhinal cortex has been suggested to store within-domain associations such as word–word associations (Mayes et al., 2007). The perirhinal cortex may have triggered the reactivation of neocortical memory traces of word pairs in the regions of prefrontal cortex that support semantic analyses and analogical reasoning (Morrison et al., 2004; Bunge et al., 2005; Green et al., 2006b, 2010). These prefrontal activations were smaller than activations in these same regions evoked in response to broken analogs. The concerned regions were the right middle and superior frontal gyrus (BA 10, 11) and the left middle frontal gyrus (BA 9, 10). These ventromedial prefrontal regions have been found to support the analogical mapping of elements of a current with elements of a past episode (Green et al., 2006b, 2010). Accordingly, unconscious memories of subliminal word pairs in the left rhinal cortex may have facilitated the processing of semantic relations between words in analogs by way of the ventromedial prefrontal cortex. Signal attenuations to analogs versus broken analogs in more lateral prefrontal regions may have facilitated the relational analysis of the two words in analogs (Blumenfeld and Ranganath, 2006; Murray and Ranganath, 2007). Further signal attenuations in a more dorsal frontal region (BA 6) suggest that the semantic retrieval was facilitated for words in analogs versus broken analogs (Binder et al., 2009). Other signal attenuations were located in the right angular and supramarginal gyri as well as the right middle and superior temporal gyri. These regions may have facilitated the detection of semantic feature overlap between encoding words (e.g., table–car) and words in analogs (e.g., desk–bus) (Patterson et al., 2007).

**Table 3:** **Retrieval of analogous semantic relations**.

Brain region	BA	Left/right	MNI-coordinates			*k*	*t*	*z*
			*x*	*y*	*z*			
**ANALOGS > BROKEN ANALOGS**								
Perirhinal cortex	35	L	−18	−32	−14	8	3.02	2.94
**BROKEN ANALOGS > ANALOGS**								
Middle frontal gyrus	10	R	26	56	14	32	3.58	3.45
Superior frontal gyrus	10	R	18	54	28	106	4.27	4.07
Middle frontal gyrus	11	R	24	46	−10	27	4.37	4.15
Medial frontal gyrus	8	L/R	−2	40	46	70	3.94	3.78
Middle frontal gyrus	47	L	−40	38	−4	25	3.67	3.54
Superior frontal gyrus	6	R	14	32	62	24	3.76	3.62
Middle frontal gyrus	9, 46	R	44	30	30	89	4.49	4.26
Middle frontal gyrus	9	L	−34	28	22	106	4.5	4.27
Inferior frontal gyrus	45	L	−48	14	20	62	3.81	3.66
Middle frontal gyrus	6	R	28	12	66	59	4.74	4.47
Middle temporal gyrus	22, 21	R	56	−40	4	236	5.7	5.27
Angular, supramarginal gyrus	39, 40	R	56	−64	36	52	3.87	3.72

**N* = 24 (good subliminal encoders); statistical maps were thresholded at *p* = 0.005, *k* = 0 in medial and anterior temporal lobe regions, all other regions *p* = 0.001, *k* = 20*.

Finally, analogs elicited less activation than broken analogs in regions supporting conflict monitoring. These conflict monitoring regions concern posterior portions of the medial prefrontal cortex (Ridderinkhof, 2004), namely the medial frontal gyrus (BA 8) and the middle frontal gyrus (BA 9). These regions in the posterior medial prefrontal cortex may have contributed to the detection of the mismatch between broken analogs and corresponding representations of encoding word pairs.

### Encoding activation predicts analogy detection

**Figure 4:**
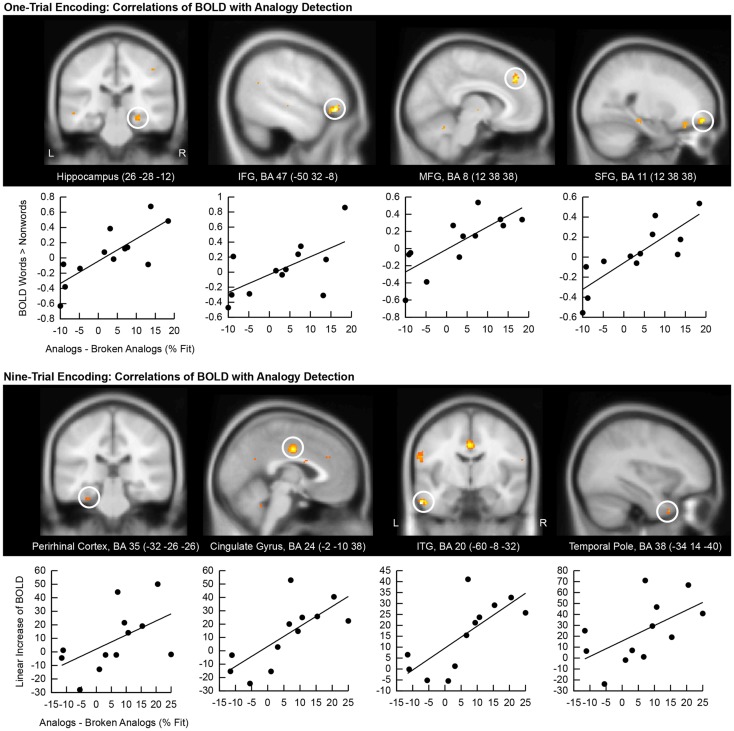
**Correlation of encoding-related brain activity with analogy detection at test**. The location of the cluster of voxels, where correlations reached significance, is shown on anatomical brain images. BA, Brodmann area; IFG, inferior frontal gyrus; SFG, superior frontal gyrus; MFG, medial frontal gyrus; ITG, inferior temporal gyrus. Scatter plots of these correlations are presented below brain images. The first eigenvariate of the cluster of significantly correlating activity is displayed in arbitrary units on the *y*-axis. The difference in the percentage of fit responses to analogs versus broken analogs is displayed on the *x*-axis. All data come from good subliminal encoders (*N* = 12). The top panel shows correlations of activity increases to subliminal word pairs versus pairs of consonant strings during one-trial encoding with analogy detection. The bottom panel shows correlations of linear activity increases over the ninefold encoding of word pairs with analogy detection.

We correlated the good subliminal encoders’ single-trial encoding contrasts (subliminal word pairs versus subliminal pairs of consonant stings) with their behavioral performance on unconscious analogy detection (difference in the percentage of fit responses given to analogs versus broken analogs). Positive correlations appeared in the right hippocampus (Figure 4, top), which confirms that the hippocampal encoding of subliminal word pairs mediated analogy detection. Another cluster emerged in the medial frontal gyrus (BA 8) suggesting that increased effort in a region supporting semantic word analysis (Binder et al., 2009) was predictive of better analogy detection at test. A further cluster was located in the superior frontal gyrus (BA 11), which – as noted earlier – has been implicated in the integration of distant semantic concepts (Green et al., 2006b, 2010). A further cluster was located in the left inferior frontal gyrus (BA 47), which has been found to support the evaluation of relationships between elements in a stimulus (Blumenfeld and Ranganath, 2006; Murray and Ranganath, 2007).

Next, we computed correlations between the good subliminal encoders’ activity increases over nine encoding trials and their performance at test (Figure 4, bottom). A steeper linear activity increase in the left perirhinal cortex predicted a larger number of fit responses to analogs than broken analogs. This result substantiates that the medial temporal lobe subserved the unconscious formation of relational memories. Furthermore, a steeper signal increase in the left temporal pole and the left inferior temporal gyrus predicted a better test performance. This result corroborates that increased neural recruitment in lexical-semantic storage sites during subliminal encoding aided later analogy detection. Finally, a steeper signal increase in the middle cingulate gyrus (BA 24) predicted a better test performance.

### Brain activation at test predicts analogy detection

**Figure 5:**
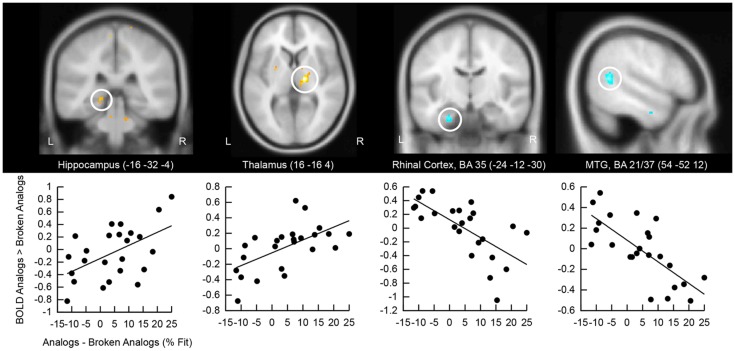
**Correlation of retrieval-related brain activity with analogy detection at test**. The location of the cluster of voxels, where correlations reached significance, is shown on anatomical brain images. BA, Brodmann area; MTG, middle temporal gyrus. Scatter plots of these correlations are presented below brain images. The first eigenvariate of the cluster of significantly correlating activity is displayed in arbitrary units on the *y*-axis. The difference in the percentage of fit responses to analogs versus broken analogs is displayed on the *x*-axis. All data come from good subliminal encoders (*N* = 24; data collapsed over Encoding Intensity).

We correlated the good subliminal encoders’ activation difference to analogs versus broken analogs with their performance at detecting analogies unconsciously (Figure 5). Positive correlations emerged in the right and left hippocampus and the right thalamus. The thalamus is a part of the episodic memory network (Winocur et al., 1984; Aggleton et al., 2011; Pergola et al., 2012). Our thalamic cluster lies in the mediodorsal nucleus of the thalamus, which has projections to the prefrontal cortex and the temporal lobe (Behrens et al., 2003). Thus, the mediodorsal nucleus of the thalamus might have mediated the interaction of medial temporal with prefrontal regions. Negative correlations were located in the left perirhinal cortex and the right middle temporal gyrus. Activity reductions in perirhinal cortex track familiarity with a stimulus (Voss et al., 2009) – in our case the familiarity with semantic relations in analogs. Activity reductions in the middle temporal gyrus likely reflect neural facilitation during the re-processing versus first-time processing of semantic relations. Familiarity and facilitated semantic processing might relate to enhanced analogy detection.

### Good subliminal encoders exhibited more activity in the medial temporal lobe than poor subliminal encoders

Comparisons between good and poor subliminal encoders underscored that the episodic memory network supported unconscious memory formation and analogy detection (Table 4). A between-group *t*-contrast of good versus poor subliminal encoders’ brain activity during one-trial encoding of word pairs revealed two significant left hippocampal clusters. This result indicates that good encoders activated the hippocampus more strongly than poor subliminal encoders in response to subliminal word pairs. Moreover, a steeper increase of the BOLD signal to repeating subliminal word pairs during nine-trial encoding was located in the left hippocampus and bilateral rhinal cortex of good versus poor subliminal encoders. Finally, good subliminal encoders activated the left hippocampus and left parahippocampal gyrus to a greater extent than poor subliminal encoders in response to analogs versus broken analogs presented at test.

**Table 4:** **Comparisons between good and poor subliminal encoders**.

Brain region	BA	Left/right	MNI-coordinates			*k*	*t*	*z*
			*x*	*y*	*z*			
**GOOD > POOR SUBLIMINAL ENCODERS, ONE-TRIAL ENCODING (WORDS > NONWORDS)**								
Middle cingulate gyrus	23	R	6	−14	30	39	4.41	3.72
Hippocampus		L	−30	−40	2	35	4.6	3.83
Hippocampus		L	−38	−20	−16	56	4.16	3.56
**GOOD > POOR SUBLIMINAL ENCODERS, NINE-TRIAL ENCODING (LINEAR INCREASE)**								
Postcentral gyrus	4	L	−60	−18	46	29	5.44	4.28
Hippocampus, rhinal cortex		L	−30	−16	−24	13	3.42	3.03
Rhinal cortex		R	26	−24	−20	23	3.64	3.19
**INTERACTION OF RETRIEVAL CONDITION WITH SUBLIMINAL ENCODING QUALITY** **(ANALOGS > BROKEN ANALOGS, GOOD > POOR SUBLIMINAL ENCODERS)**								
Parahippocampal gyrus		L	−20	−32	−16	51	3.9	3.74
Hippocampus		L	−40	−32	−4	19	3.26	3.17

**N* = 51; statistical maps thresholded at *p* = 0.005, *k* = 0 in medial and anterior temporal lobe regions, all other regions *p* = 0.001, *k* = 20*.

## Discussion

We report evidence for the unconscious detection of analogical relations in a present and a past situation. Participants encoded subliminal word pairs and later judged the semantic fit of new words in supraliminal pairs that either retained a previously encoded semantic relation (analogs) or not (broken analogs). The successful unconscious detection of semantic relations in supraliminal test words that were analogous to semantic relations in subliminal encoding words was suggested by a larger number of fit responses given to analogs than broken analogs at test. Hence, episodically related versus unrelated words in test pairs were more often judged as closely related semantically. In other words, semantic relations encoded in an unconsciously experienced episode had intruded into judgments of semantic distance made at test. This effect corresponds to findings from supraliminal, i.e., conscious, stimulus processing. When two unrelated words, which had been presented in the same encoding context and were therefore episodically (but not semantically) related, were represented at test, they appeared closer semantically than words that had not been presented in the same encoding context; or they appeared equally close as words that were related semantically (McKoon and Ratcliff, 1979, 1986; Dosher and Rosedale, 1991; Patterson et al., 2009; Coane and Balota, 2011). This line of research suggests that connections between mental representations or between nodes in the semantic network, which have been co-activated in the same encoding context, acquire a greater linkage strength leading to the impression of stronger conceptual relatedness. The co-occurrence of concepts in naturalistic events is indeed one way how the semantic system may be dynamically (re)organized throughout life (Coane and Balota, 2011). Although test pairs in the current study did not contain the subliminal encoding words but semantic neighbors thereof, the same principle seems to apply.

These modifications in the semantic system apparently relied on the relational binding of subliminal words in the hippocampus. The hippocampus is thought to assist the encoding of events by association formation between simultaneously activated areas of the neocortex (Teyler and DiScenna, 1986; Treves and Rolls, 1994). Because our task required a rapid relational encoding process with resulting flexible representations of word pairs (Cohen and Eichenbaum, 1993; Henke, 2010), we had hypothesized a role for the hippocampus in relational encoding and retrieval. Our neuroimaging results confirmed the expected involvement of the hippocampus and other structures of the episodic memory network during subliminal encoding and during unconscious retrieval in the test situation.

The current results emphasize the intimate relationship between memory and analogical reasoning. While previous neuroimaging studies of analogical reasoning made use of pre-existing knowledge stored in semantic memory that acted as source knowledge (Bunge et al., 2005; Green et al., 2006b, 2010), we aimed at the unconscious detection of analogies to episodic rather than semantic memories. Therefore, we had participants establish new episodic source knowledge in the experimental session. Due to our particular interest in unconscious analogical reasoning, we excluded conscious awareness already at the time of encoding the source episodes. Our finding of a successful unconscious detection of analogies between current and past episodes connects with purely behavioral studies of unconscious analogical reasoning, in which participants also encoded source information – although supraliminal source information – in the experimental session itself (Schunn and Dunbar, 1996; Day and Gentner, 2007; Day and Goldstone, 2011). The source knowledge gained in these studies was of a procedural (Day and Goldstone, 2011) or episodic nature (Schunn and Dunbar, 1996; Day and Gentner, 2007). Because all encoding material was presented suprathreshold for conscious inspection, information about conscious awareness of insight into analogies had to be determined with post-experimental questionnaires. This procedure is liberal because a potential conscious detection of analogies of a current with a past (conscious) learning situation may remain unreported. The subliminal presentation of the source information excludes consciousness of analogy detection more rigorously. That the presentation of encoding word pairs was indeed subliminal was demonstrated in our study by the participants’ chance performance on the direct test of awareness (Greenwald et al., 1996; Snodgrass and Shevrin, 2006).

In line with studies of *conscious* analogical reasoning (Bunge et al., 2005; Green et al., 2006b, 2010; Speed, 2010; Knowlton et al., 2012), the largest signal change during *unconscious* analogy detection (analogs versus broken analogs) was located in the medial prefrontal cortex. Ventromedial (BA 10, 11) prefrontal areas are thought to promote the integration of distant semantic concepts as a means to achieve analogical mapping (Bunge et al., 2005; Green et al., 2006b, 2010). Perceived semantic distance is likely represented in medial prefrontal cortex because ventromedial prefrontal activity scaled positively with the semantic distance between two words in a pair, as reported in a study of conscious analogical reasoning (Green et al., 2010). In the present study, ventromedial prefrontal activity was reduced in response to analogs versus broken analogs, which corresponds to the subjective decrease in semantic distance of words in analogs versus broken analogs (see also Reber and Henke, 2011). At the intersection of memory and decision making, the ventromedial prefrontal cortex brings together the contents of long-term memory provided by the hippocampus and the planning abilities of prefrontal cortex to guide behavior (Euston et al., 2012; Guitart-Masip et al., 2013). We assume that the display of analogs in the test situation had triggered the reactivation of unconscious memories of subliminal word pairs through the medial temporal lobe, which in turn evoked a reinstatement of activity within the medial prefrontal cortex and other neocortical regions (McClelland et al., 1995). This reinstatement may have facilitated the processing of semantic relations between words in analogs.

The current findings show that the medial temporal lobe including hippocampus mediated unconscious relational encoding and the flexible retrieval of stored relations in the test situation, which enabled the unconscious detection of analogous relationships. Because the study format (e.g., table–car) differed from the test format (e.g., desk–bus), relational memories must have been expressed flexibly in the test situation. Hence, our data suggest that the encoding of new semantic relations and their flexible expression do not depend on conscious awareness of encoding and retrieval. This finding supports our notion that hippocampus-dependent relational encoding and retrieval may proceed with and without conscious awareness of encoding/retrieval (Henke, 2010) and questions views that link hippocampal processing and episodic memory to consciousness (Tulving, 1985; Reber and Squire, 1994; Squire and Zola, 1996; Squire et al., 2007; Moscovitch, 2008). The current and past findings of unconscious relational encoding (Henke et al., 2003a,b; Degonda et al., 2005; Duss et al., 2011; Reber and Henke, 2011, 2012; Reber et al., 2012) and past findings of unconscious relational retrieval (e.g., Greene et al., 2001; Leo and Greene, 2008; Hannula and Ranganath, 2009) are better accommodated by a memory model that divides between memory systems based on processing modes rather than consciousness (Henke, 2010).

Beyond memory, our results highlight the extent to which purely unconscious processes may contribute to higher-order cognition. Our study adds to a growing body of literature that pushes the boundaries of unconscious cognition into fields such as cognitive control (Van Gaal et al., 2008, 2010), decision making (Pessiglione et al., 2007, 2008), and free will (Soon et al., 2008). It becomes increasingly clear that our behavior and our conscious thoughts are deeply influenced by unconscious processes.

## Author Contributions

Thomas P. Reber designed and conducted the research, analyzed the data, and wrote the paper. Roger Luechinger and Peter Boesiger provided MR-Technology and infrastructure. Katharina Henke designed the research, analyzed the data, and wrote the paper.

## Conflict of Interest Statement

The authors declare that the research was conducted in the absence of any commercial or financial relationships that could be construed as a potential conflict of interest.

## Footnotes

^1^http://www.neurobs.com/presentation

^2^http://www.fil.ion.ucl.ac.uk/spm/
